# Can the assessment of skin injuries and keel bone damage at the slaughterhouse replace on-farm assessments?

**DOI:** 10.1371/journal.pone.0309137

**Published:** 2024-12-02

**Authors:** Lisa Jung, Boris Kulig, Helen Louton, Ute Knierim

**Affiliations:** 1 Farm Animal Behaviour and Husbandry Section, University of Kassel, Kassel, Germany; 2 Agricultural and Biosystems Engineering, University of Kassel, Kassel, Germany; 3 Faculty of Agricultural and Environmental Sciences, Animal Health and Animal Welfare, University of Rostock, Rostock, Germany; Nasarawa State University, NIGERIA

## Abstract

Two major welfare problems in laying hen farming are keel bone damage (KBD) and cannibalism. Their assessment is time-consuming, needs well-trained assessors, and prevalence estimates are often imprecise due to small sample sizes. Here, the bottleneck slaughterhouse comes into focus where large numbers of animals can be inspected. However, this is only an option if the prevalences recorded at the slaughterhouse reasonably agree with on-farm assessments. The aim of this study was to compare the prevalence of KBD and skin injuries in 20 commercial laying hen flocks (i) before depopulation on-farm (ii) after transport and lairage time at arrival at the slaughterhouse, and (iii) at the slaughter line. Bland-Altman plots and equivalence tests were conducted. In addition, the consistency of welfare evaluations of the results according to a traffic light scheme was investigated. Cloacal injuries could not technically be recorded on the slaughter line. With an arbitrary precision of ± 2% for dorsal skin injuries and ± 5% for KBD, the results from farm and slaughter line did not reach equivalence. For dorsal skin injuries, the detected mean prevalence across all flocks examined increased numerically from the farm (15.1%) to the slaughter line (22.8%). In addition, the traffic light evaluations changed between farm and slaughter line in 80% of cases in different directions. Therefore, it cannot be recommended to derive evaluations of on-farm welfare from assessments of skin injuries at the slaughter line. In contrast, the mean detected prevalence of KBD across all flocks decreased consistently (r = 0.794) from the farm (56.0%) to the slaughter line (41.7%). It can be concluded that the assessment of KBD at the slaughter line consistently underestimates KBD prevalences compared to on-farm assessments, but this can be taken into account in the interpretation of the results. Slaughter line assessment of KBD may be a feasible option for monitoring severe welfare problems due to KBD in commercial practice.

## Introduction

Societal concerns about the welfare of laying hens relate mainly to the use of cage systems and the practice of beak trimming. This is reflected in the recent European Citizens’ Initiative ‘End the Cage Age’ [1] and, for instance in Germany’s ban on all cages for laying hens by 2028 at the latest [2] and the voluntary agreement not to perform beak trimming in laying hens since 2016 [3]. However, these practices can also be associated with serious welfare problems that need to be urgently addressed. Two of the main problems are considered to be keel bone damage (KBD), particularly fractures [4], and cannibalism [5].

KBD includes deviations or fractures or both. They can be found in all housing systems and across all genetics [6]. Multiple factors contribute to the prevalence of KBD, related to genetics and laying performance as well as management, nutrition and housing during rearing and the production period [7–9]. Keel bone fractures can lead to pain [10], reduced mobility [7], and decreased laying performance [11], while the welfare implications of mere keel bone deviations are less clear. However, depending on the assessment method, it is not always possible to clearly differentiate between fractures and deviations [9]. According to Scholz et al. [12] most deviations are accompanied or caused by fractures. Therefore, when using methods like visual assessment and palpation, the absence of detectable callus or dislocation does not prove the absence of fractures, but highly deviated keel bones are likely to be fractured [12, 13]. Only with the use of histology [12] or radiology [14] can a clear distinction be made. As fractures and mere deviations can have different causes, such differentiation is mostly advantageous or necessary for scientific investigations [15]. However, they are not suitable for on-farm welfare assessment and mostly not necessary if the aim is to indicate whether or not there is a problem. Moreover, as the proportion of fractures is typically high in keel bone damage [16], for practical reasons differentiation is often not a prerequisite for taking action against KBD. In general, however, any method of assessment other than histology or radiology is likely to underestimate the prevalence of KBD, namely fractures [14, 17, 18].

Cannibalism can be identified by direct observation of the behavior of the hens, as well as by the recording of injuries, especially on the back and in the cloaca region of the victims [19]. The latter is a more feasible method for on-farm welfare assessment than behavioral observations. Again, multiple risk factors related to genetics, rearing, management including the keeping of hens with intact beaks (11), nutrition and housing can increase the risk of a feather pecking outbreak [20] with possibly subsequent cannibalism. However, cannibalism directed at toes or the cloaca can develop independently from feather pecking [21, 22]. Cannibalism is an indication of failed coping in the active bird and leads to pain and increased risks of disease and death in the victims [23].

Laying hen farmers are often unaware of the extent of these welfare problems in their flocks because the handling and reliable assessment of a sufficient number of animals by visual inspection and palpation is challenging, especially for KBD. Nonetheless, more and more, retailers and, in some countries like Germany, welfare law [2] require welfare monitoring through an assessment of animal-based indicators. Several animal welfare protocols to be implemented on-farm include KBD and skin injuries as suitable welfare indicators for laying hens [24, 25]. In search of less time-consuming assessment methods, the bottleneck slaughterhouse comes into focus. At slaughter, a large number of animals or even the whole flock can be easily inspected [26]. However, a challenge for assessments at the slaughterhouse may be frequent change of personnel which makes it difficult to provide proper training. In addition, a prerequisite is a reasonable match between detected prevalences on-farm and at the slaughterhouse. To our knowledge this has not been investigated yet. Grafl et al. [27] assessed selected welfare measures in laying hens from 79 flocks at the slaughter line and recorded them as part of a novel scoring system, but without verifying their concordance to prevalences on-farm. Until now there is no internationally published study available that compares prevalences assessed at the slaughterhouse and on-farm. Deviations between results from farm or the slaughter line may be caused by injury during catching or transport or by differing assessments in live birds or at the slaughter line. Therefore, assessments at three different points should be investigated: (i) at the end of lay before depopulation on the farm as a reference, (ii) after transport and lairage time at arrival at the slaughterhouse to check for possible effects of catching or transport, and (iii) at the slaughter line. As a minimum requirement, the deviation between detected prevalences on-farm and at the slaughter line should not affect the welfare evaluation of the assessment results, e.g. being in the ‘target range’, ‘early warning range’ or ‘alarm range’. Such a ‘traffic light approach’ has been developed in Germany in recent years with the involvement of a broad number of experts (28). This evaluation framework for farmers sets limits for the different ranges. The ‘alarm range’ is intended to indicate approaching and serious welfare problems at flock level which should prompt the farmer to take action in the short or longer term [28].

Our aim was to evaluate whether the assessment of keel bone damage and skin injuries at the slaughterhouse provides similar information to that by on-farm welfare assessment and could therefore be an option to replace the time-consuming on-farm scoring of live birds. This involved several steps: (i) to determine the inter-observer reliability (IOR) of the different scoring systems, including trained and untrained observers, (ii) to compare the estimates of detected flock prevalences at the different assessment points with regard to their equivalence, taking into account the effects of imprecision in prevalence estimates due to differing sample sizes, (iii) to check the concordance in the welfare evaluation of the detected flock prevalences on-farm and at the slaughter line. In addition, in a sub-sample we examined the accuracy of the KBD assessment by visual inspection of the carcass by reference to the dissected bones.

Our hypotheses were that recorded prevalences of skin injuries would increase on average from farm to arrival to the slaughter line due to additional injury during the process, but that the detected KBD prevalences would be similar on-farm and at arrival, whereas they would be lower at the slaughter line where only visual assessment without palpation is feasible.

## Animals, materials and methods

The hens in this study did not undergo any experimental procedure and were only monitored by careful, non-invasive clinical scoring which is also required by animal welfare law for the welfare assessment of farm animals. The birds were reared and kept for production for human consumption according to national law and guidelines, and not for experimental purposes. Therefore, ethical approval was waived for this study, as approved by the Designated Veterinarian for Institutional Animal Care at the University of Kassel.

### Animals

The study took place in the years 2017 to 2019 in 20 commercial laying hen flocks and at a poultry slaughterhouse in Germany. Different non-cage housing systems and birds from different genetics were included (Table 1). All hens had intact beaks.

**Table 1:** Characteristics of the investigated laying hen flocks with numbers of assessed hens on-farm concerning skin and keel bone status.

Flock-number	Genotype	Housing system	Free-range	Week of age	Flock size (N)	No. of hens assessed on-farm
1	Novogen	Multi-tier	Yes	72	1,140	100
2	ISA Brown	Floor	Yes	76	2,550	360
3	LB	Multi-tier	Yes	68	3,000	315
4	LB	Floor	Yes	72	3,000	360
5	LSL/LB	Multi-tier	No	91	900	360
6	LB	Multi-tier	Yes	86	2,737	360
7	LB	Multi-tier	No	72	n.a.	360
8	LB	Multi-tier	Yes	72	n.a.	360
9	LB	Multi-tier	Yes	85	2,400	307
10	Brown Nick	Multi-tier	Yes	104	3,000	225
11	Novogen	Multi-tier	Yes	84	4,200	180
12	LB	Multi-tier	Yes	72	n.a.	100
13	LB	Floor	Yes	70	3,256	250
14	LB	Multi-tier	Yes	82	2,680	234
15	LB	Floor	No	72	880	360
16	LB	Floor	Yes	68	2,880	360
17	LB	Mobile housing	Yes	74	n.a.	360
18	LSL	Multi-tier	No	63	10,000	150
19	LB	Floor	Yes	83	1,200	360
20	LB	Floor	Yes	80	n.a.	334

LB = Lohmann Brown, LSL = Lohmann Selected Leghorn, n.a. = no information available

### Scoring schemes and inter-observer reliability (IOR) tests

The assessment of the condition of the keel bones and skin of the birds or carcasses followed the scoring schemes shown in Table 2 [29]. Slightly different scoring schemes had to be used for the assessment of KBD on-farm and at arrival (palpation and visual inspection; three-point scale) and at the slaughter line (only visual inspection; two-point scale).

**Table 2:** Scoring schemes for the assessment of skin and keel bone status in laying hens^1^ [29].

**Score**	**Definition of skin status on-farm, at the arrival and at the slaughter-line**
0	No injury
1	Maximum of 2 injuries of ≤ 1 cm longest diameter (fresh or crusted)
2	3 or more injuries ≤ 1 cm, or 1 injury > 1 cm longest diameter (fresh or crusted)
**Score**	**Definition of keel bone scores for live animals; palpation and visual inspection^2^**
0	Intact, no palpable or visible callus or dislocation, and no lateral, dorsal, or ventral deviations from straight axis
1	Palpable or visible callus or dislocation, or lateral, dorsal, or ventral deviations from straight axis ≤ 1 cm
2	Palpable or visible callus or dislocation, or lateral, dorsal, or ventral deviations from straight axis > 1 cm
**Score**	**Definition of keel bone scores for carcasses; visual inspection only**
0	Intact, no deviation from straight axis in any direction due to visible dislocation or deformity and no visible callus
1	Visible dislocation or callus, or lateral, dorsal, or ventral deviation from straight axis

^1^ the keel bone tip (last 1,5 cm caudal part of the bone) was separately assessed as intact/damaged, but not included in the analyses

On-farm IOR tests were carried out as follows: one observer carefully and unselectively caught a hen from different barn areas, and after scoring passed them on to the next observer. Each observer recorded the scores individually and independently. In the IOR tests, the first author always acted as expert (silver standard) paired with either a trained or untrained further assessor. IOR for skin injuries and KBD were carried out separately. IOR of skin injury assessment was tested ten times on-farm with 30 to 110 hens each time (660 hens in total). Two trained and one untrained person were involved. IOR of KBD assessment was tested 14 times on-farm with 20 to 80 live hens each time (681 hens in total), with one untrained and four trained persons. The trained assessors had been instructed by the expert and had already scored at least 150 hens using the current scoring scheme, whereas the untrained persons had scored less than 150 hens to no hen. The IOR tests were carried out in different flocks with different genotypes. For a better view of the skin injuries, the observers wore headlamps.

IOR tests at the slaughter line were carried out after defeathering. The assessors stood close together about 30 cm in front of the slaughter chain. One person, who was not involved in the IOR testing, stood further in front of the chain and arbitrarily indicated the hen to be assessed using a pointer so that all persons always assessed the same hen. IOR for dorsal skin injuries were performed two times at the slaughter line on 40 and 79 carcasses (see Table 3) with two observer pairs including two untrained persons. At the slaughter line, 14 IOR tests were performed for KBD assessment on 46 to 400 carcasses (in total 2273 carcasses, see Table 4). In these IOR-tests, eight observer pairs with four trained and four untrained people were included.

**Table 3:** Results of inter-observer reliability tests (prevalence-adjusted bias-adjusted kappa: PABAK) for the assessment of skin injuries in laying hens on farm and at the slaughter line using a three-point scale; each line represents the result of an observer pair of the expert (silver standard) with either an experienced or inexperienced assessor.

N of hens	PABAK	Body-Area	Place	Experience^1^
30	0.75	Cloaca	On-farm	Yes
110	0.84	Cloaca	On-farm	Yes
110	0.88	Cloaca	On-farm	Yes
30	0.95	Cloaca	On-farm	No
30	0.75	Back	On-farm	Yes
30	0.75	Back	On-farm	Yes
110	0.80	Back	On-farm	Yes
110	0.82	Back	On-farm	Yes
30	1	Back	On-farm	No
50	1	Back	On-farm	No
79	0.87	Back	Slaughter line	No
40	0.95	Back	Slaughter line	No

^1^ experience of the assessor: yes = person who had scored skin injuries of more than 150 hens in at minimum two sessions, no = person who had previously scored skin injuries in less than 150 hens

**Table 4:** Results of inter-observer reliability tests (prevalence-adjusted bias-adjusted kappa: PABAK) for the assessment of keel bone damage in laying hen carcasses by visual inspection; each line within ‘IOR on-farm’ and ‘IOR slaughter line’ represents the result of an observer pair of the expert (silver standard) with either an experienced or inexperienced assessor on-farm or at the slaughter line.

IOR on-farm				IOR slaughter line		
**N hens**	**PABAK**		**Experience** ^1^	**N hens**	**PABAK**	**Experience** ^**1**^
	**3-point scale**	**2-point scale**			**2-point scale**	
62	0.71	0.94	Yes	100	0.90	Yes
30	0.75	0.93	Yes	76	0.82	Yes
80	0.68	0.83	Yes	76	0.82	Yes
30	0.60	0.80	Yes	157	0.81	Yes
50	0.64	0.73	Yes	110	0.78	Yes
62	0.52	0.58	Yes	400	0.77	Yes
62	0.42	0.55	No	90	0.76	Yes
45	-	0.64	Yes	90	0.73	Yes
32	-	0.69	Yes	68	0.73	Yes
30	-	0.69	Yes	400	0.67	Yes
77	-	0.79	Yes	46	0.57	Yes
20	-	0.80	Yes	110	0.78	No
27	-	0.56	No	150	0.75	No
74	-	0.57	No	400	0.65	No

^1^experience of the further assessor: yes = person who had scored KBD of more than 150 hens in at minimum two sessions, no = person who scored KBD in less than 150 hens

Additionally, 128 hens from three different flocks were taken from the slaughter line after evisceration and defeathering and individually marked with a number. The keel bones of these haphazardly selected hens were assessed via visual inspection of the carcasses. The keels were then dissected from the carcass and later placed in a hot water bath at 70°C with in relation 1:10 bioenzyme:water for 12 hours, resulting in pure bones that were sanitized using an aqueous solution with 1% H_2_O_2_ for 3 hours. The assessment of the bones was carried out with the same scheme as shown in Table 2.

### Data collection on farm and at the slaughterhouse

In each of the 20 participating flocks, the first assessment took place at the farm on the day of depopulation, or in two cases (flock 7 and 8, Table 1) one day before. Samples of 100 to 360 hens per flock were haphazardly caught by two trained persons from different barn areas, the covered veranda and free range, and assessed for KBD and skin injuries by palpation and visual inspection, respectively. To avoid double scoring, hens received a small blue ink mark at the inner leg after scoring which faded within some hours. The barns were darkened to make catching less stressful. The second assessment of the same flock took place at the arrival at the slaughterhouse. Palpation and visual inspection were conducted each time by the same assessor (one of two trained assessors), taking hens haphazardly out of the transport crates. Therefore, not necessarily the same individuals were assessed in the first and second scoring and the prevalences relate to the flock, not to the individuals. A second person assisted in noting the scores. As many hens as possible (minimum 31, maximum 356 hens per flock) were scored before slaughter without unduly delaying the process. After scoring, each hen was handed over to a slaughterhouse employee for shackling. The hens were then stunned in an electrical bath and slaughtered by bleeding. The slaughterhouse was EU approved.

**Fig 1:**
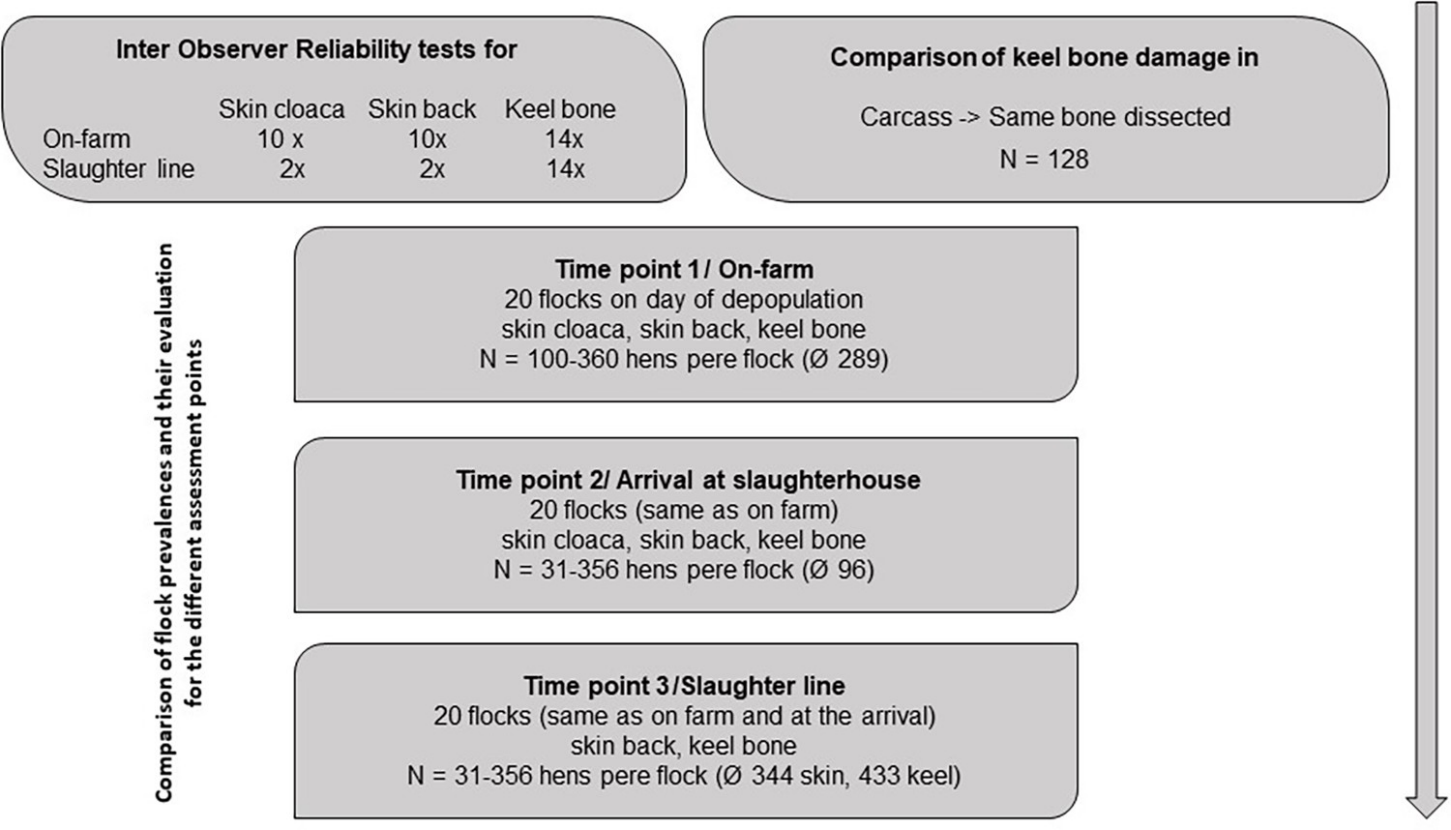
Sequence of data collection for the analysis of reliability testing and prevalence estimation of skin injuries and keel bone damage at the three assessment points on-farm, arrival and slaughter line.

The third assessment was conducted after killing and defeathering at the slaughter line by the same assessors as before. Keel bone and skin status were scored on the hanging carcasses in the moving shackles by visual inspection only. One assessor scored skin injuries, another KBD. As many hens as possible were scored haphazardly (skin injuries minimum 41, maximum 808 hens; KBD minimum 48, maximum 631 hens per flock). The speed of the slaughter line was reduced to 30 hens/min for this purpose. Due to the high position of the hens at the slaughter line, it was not possible to assess skin injuries at the cloaca. Therefore, skin injuries at the cloaca were only evaluated on-farm and at arrival. Fig 1 shows the single steps of data collection.

### Statistics

Inter-observer reliability was evaluated pairwise between silver standard and each trained or untrained assessor using the Prevalence-Adjusted Bias-Adjusted Kappa (PABAK) in SPSS 24: PABAK = ((k*p)-1)/(k-1), with k = number of categories, p = proportion of matchings between assessors. Results were interpreted based on Landis and Koch [30] and Gunnarsson [31] as follows: ≤ 0.40: not acceptable; 0.41–0.60: acceptable; 0.61–0.80: substantial; 0.81–1.00: almost perfect.

Accuracy, precision, sensitivity and specificity of the detection of keel bone damage by visual inspection of the carcass in relation to the inspection of the dissected bone were determined on the basis of a confusion matrix and using the following formulae:

$$Precision=\frac{TP}{TP+FP}$$

$$Accuracy=\frac{TP+TN}{TP+TN+FP+FN}$$

$$Sensitivity=\frac{TP}{TP+FN}$$

$$Specificity=\frac{TN}{TN+FP}$$

with TP = true positives, TN = true negatives, FP = false positives, FN = false negatives.

For skin injuries and keel bone damage, flock prevalences for the different assessment points and the corresponding confidence intervals for the given flock and sample sizes were calculated according to Hedderich and Sachs [32]:

$$\pi_{upper}\approx \hat{p}+Z_{\left(1-\frac{\alpha }{2}\right)}\times \sqrt{\frac{\hat{p}(1-\hat{p})}{n}\times (\frac{N-n}{N-1})}$$

$$\pi_{lower}\approx \hat{p}-Z_{\left(1-\frac{\alpha }{2}\right)}\times \sqrt{\frac{\hat{p}(1-\hat{p})}{n}\times (\frac{N-n}{N-1})}$$

with π = true prevalence of the flock, $\hat{p}$ = estimated prevalence, Z = degree of confidence, *α* = 95%, N = flock size, n = sample size.

Using the Software JMP 15 [33], an equivalence test was conducted for the comparison of prevalences recorded on-farm and at slaughterhouse arrival, or on-farm and at the slaughter line. Zones of equivalence (defined by the lower and upper equivalence limit) were arbitrarily set at ± 2%-points for skin injuries and ± 5%-points for KBD as highest tolerable difference. Equivalence was examined using the Two One-Sided T-test (TOST). Results are presented in Bland Altman plots and the correlation between assessments is stated.

Moreover, the flock prevalences were allocated to a target (green), early warning (yellow) or alarm (red) range according to the German evaluation framework [28]. The framework implies the following reference values for skin injuries (score 1 and 2): ≤ 2.0%, 2.1–29.9%, ≥ 30.0%, and for KBD (score 1 and 2): ≤ 10.0%, 10.1–45.9%, ≥ 46.0%. The number of deviations in these evaluations between assessments on-farm and arrival at the slaughterhouse, as well as between on-farm and at the slaughter line were expressed as percentages of all assessments.

## Results

### Inter-observer reliability of skin lesion and keel bone damage assessments

IOR of skin lesion assessments was substantial to almost perfect with PABAK values from 0.75 to 1 (mean 0.87, median 0.87); they ranged between 0.75 and 1.00 (mean 0.87, median 0.84) on-farm, and between 0.87 and 0.95 (mean 0.91, median 0.91) at the slaughter line (see Table 3).

Reliability of the assessments of keel bone status depended on the experience of the assessors (Table 4) and was never substantial or almost perfect for palpation of live hens if an inexperienced assessor was involved. In contrast, the agreement of trained assessors ranged from 0.52–0.75 (mean 0.65, median 0.66) for the three-point scale assessment, and for the two-point scale assessment from 0.58 to 0.94 (mean 0.77, median 0.79) which was in the substantial to almost perfect range, except for one outlier. IOR for visual inspection of the carcass was always acceptable to almost perfect (carcass: 0.57–0.90, mean 0.75, median 0.77), irrespective of the experience.

### Accuracy of the visual keel bone assessment in the carcass in relation to the dissected bone

Of the 128 keel bones, 70 were classified as damaged in both carcass and dissected bone (TP) and 23 as undamaged (TN). In 30 cases, KBD was not detected in the carcass but in the dissected bone (FN). There were 5 cases of KBD detected in the carcass, but not confirmed in the dissected bone (FP). The results were 72.66% accuracy, 93.33% precision, 70.00% sensitivity and 82.14% specificity.

### Skin lesion and keel bone damage prevalences at the different assessment points

**Fig 2:**
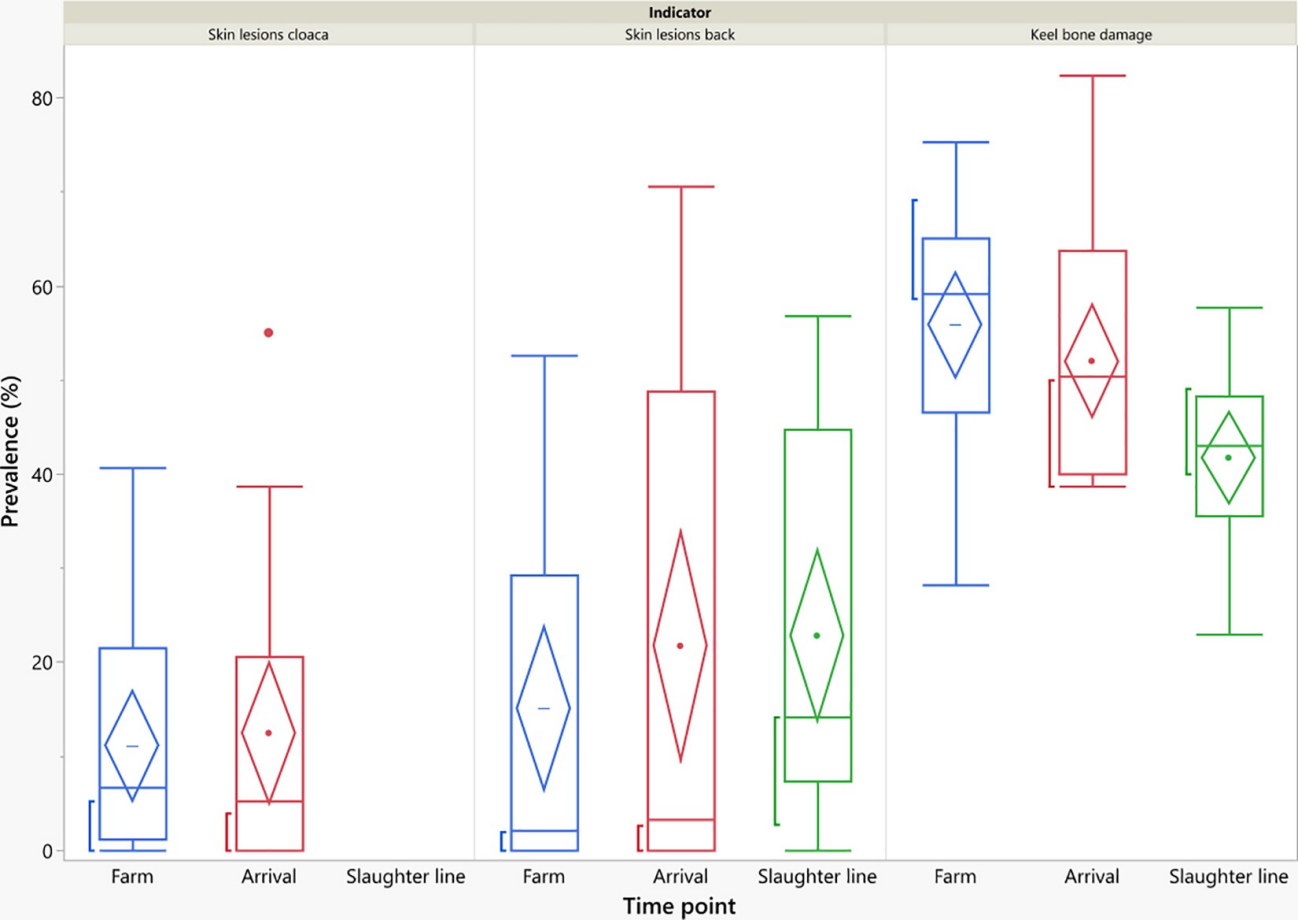
Prevalences of skin injuries and keel bone damage assessed on-farm, at the arrival and at the slaughter line.

Prevalences of skin injuries on farm varied between flocks from 0% to 40.6% at the cloaca (mean: 11.1%, median: 6.6%) and from 0% to 52.7% (mean: 15.1%, median: 2.1%) on the back. On average, prevalences of dorsal injuries numerically increased from farm (15.1%) to arrival (21.7%) and to the slaughter line (22.8%; see Fig 2).

In the on-farm assessment no flock without KBD was found, with 28.2% of hens affected in the flock with the lowest proportion, and 75.3% in the flock with the highest prevalence. On average, detected prevalences of KBD numerically decreased from farm (56.0%) to arrival (52.0%) to slaughter line (41.7%; Fig 2).

In 65% of cases, recorded prevalences for skin injuries at the cloaca on-farm and at the slaughterhouse arrival fell into the same range of the traffic light evaluation; for skin injuries at the back these were 60% and for KBD 70% (Table 5). Comparing evaluations of the on-farm versus slaughter line recordings, 20% were consistently in the same welfare range for skin injuries at the back and 70% for KBD (Table 5). However, taking account of the confidence intervals that indicate the uncertainty of the prevalence estimate, the flock prevalences of skin injuries at the cloaca assessed on-farm or slaughterhouse arrival could fall into a different evaluation range.

**Table 5:** Prevalences of skin injuries at the cloaca and at the back and of keel bone damage in 20 laying hen flocks assessed on-farm, at the slaughterhouse arrival and at the slaughter line ± confidence intervals based on sample and flock sizes, and evaluated as either being in the target area (green), early warning area (yellow, skin injuries: 2.1–29.9%, KBD:, 10.1–45.9%) or alarm area (red), according to KTBL and University of Kassel [28]. Sample sizes are shown in the S1 File.

	Prevalence (%) ±confidence interval (at a confidence level of 95%)							
**Flock**	**Skin injuries cloaca**		**Skin injuries back**			**Keel bone damage**		
	Green:≤ 2.0, red: ≥ 30.0		Green:≤ 2.0, red: ≥ 30.0			Green:≤ 10.0, red: ≥ 46.0		
	**On farm** ^**1**^	**Arrival**	**On farm** ^**1**^	**Arrival**	**Slaughter line**	**On farm** ^**1**^	**Arrival**	**Slaughter line**
1	0.00 ±0	0.00 ±0	2.00 ±2.62	1.00 ±1.86	26.83 ±13.32	60.61 ±9.15	64.30 ±9.87	47.90 ±13.84
2	1.11 ±1.00	0.00 ±0	2.22 ±1.41	0.00 ±0	0.00 ±0	53.61 ±4.78	41.30 ±10.01	41.30 ±4.94
3	40.63 ±5.13	55.00 ±12.46	26.35 ±4.60	48.33 ±12.52	52.48 ±2.94	68.30 ±4.86	70.00 ±11.48	49.10 ±4.32
4	8.06 ±2.64	2.22 ±3.00	41.94 ±4.78	48.89 ±10.17	54.09 ±5.83	62.22 ±4.70	50.00 ±10.18	46.70 ±5.21
5	12.22 ±2.62	11.76 ±10.63	52.65 ±4.00	70.59 ±15.03	56.83 ±7.58	75.30 ±3.45	82.40 ±12.56	57.70 ±2.43
6	5.28 ±2.15	3.96 ±3.73	0.00 ±0	0.00 ±0	13.48 ±4.85	69.20 ±4.45	67.30 ±8.98	48.10 ±4.49
7	30.60 ±4.25	38.70 ±17.00	28.00 ±4.14	64.50 ±16.70	14.07 ±4.55	63.10 ±4.45	38.70 ±17.00	48.40 ±3.49
8	0.83 ±0.85	16.67 ±9.29	23.33 ±3.95	50.00 ±12.46	46.06 ±7.33	59.70 ±4.58	51.70 ±12.45	34.50 ±3.23
9	2.93 ±1.76	0.00 ±0	0.00 ±0	0.00 ±0	24.21 ±5.00	49.50 ±5.22	39.70 ±12.44	23.00 ±3.18
10	28.44 ±5.67	21.90 ±6.77	30.67 ±5.80	37.96 ±7.94	45.60 ±3.99	65.80 ±5.96	39.40 ±8.00	55.40 ±4.06
11	0.00 ±0	0.00 ±0	0.00 ±0	0.56 ±0.74	2.69 ±1.06	43.30 ±7.08	45.80 ±4.95	39.10 ±4.10
12	8.00 ±5.23	0.00 ±0	50.00 ±9.64	22.00 ±10.47	14.10 ±4.67	41.00 ±9.48	50.80 ±12.63	38.60 ±3.69
13	1.60 ±1.50	6.54 ±4.61	0.00 ±0	0.00 ±0	8.48 ±2.55	61.20 ±5.81	56.10 ±9.25	46.70 ±3.85
14	3.42 ±2.23	0.00 ±0	0.00 ±0	0.00 ±0	7.25 ±3.39	28.20 ±5.51	40.20 ±10.45	25.20 ±4.76
15	23.06 ±3.35	34.67 ±10.31	0.56 ±0.59	4.00 ±4.24	18.53 ±3.98	66.10 ±3.76	65.30 ±10.31	41.90 ±5.40
16	10.00 ±2.90	10.53 ±9.70	1.94 ±1.33	2.63 ±5.06	7.60 ±1.90	45.60 ±4.81	39.50 ±15.44	25.30 ±3.00
17	1.39 ±1.13	0.00 ±0	0.00 ±0	0.00 ±0	4.57 ±3.00	38.40 ±4.71	40.00 ±8.41	25.80 ±3.75
18	0.00 ±0	0.00 ±0	0.67 ±1.29	0.00 ±0	6.27 ±2.69	55.30 ±7.90	53.10 ±13.94	44.10 ±4.33
19	26.67 ±3.82	15.77 ±3.92	29.72 ±3.95	56.54 ±5.34	42.00 ±2.65	58.60 ±4.26	61.90 ±5.23	55.20 ±6.11
20	17.07 ±3.43	30.77 ±12.78	11.68 ±2.93	26.92 ±11.80	10.37 ±2.10	52.40 ±4.55	42.30 ±13.14	40.00 ±3.03

^1^Score 1+2 combined

Flock prevalences correlated between the assessments on-farm and at the slaughterhouse arrival (skin injuries at the cloaca: r = 0.886, skin injuries on the back: r = 0.849, KBD: r = 0.665, N = 20), as well as between on-farm and at the slaughter line (skin injuries on the back: r = 0.708, KBD: r = 0.794, N = 20).

**Fig 3:**
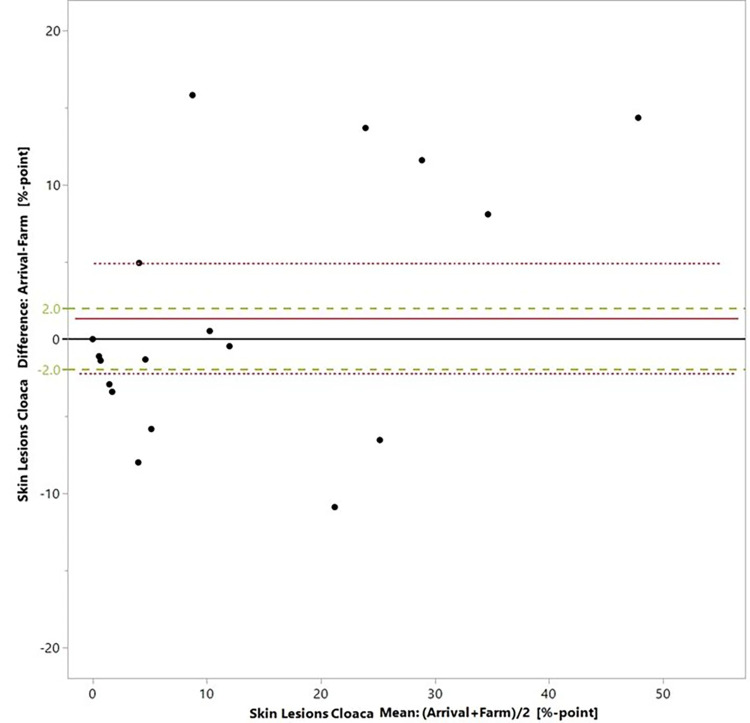
Prevalences of skin injury at the cloaca region assessed on-farm versus at the slaughterhouse arrival. The black line is the zero line, the red line is the mean difference between the prevalences, the dotted red lines delimit the confidence interval of the differences and the green lines delimit the zone of equivalence, here set at +/- 2%-points.

**Fig 4:**
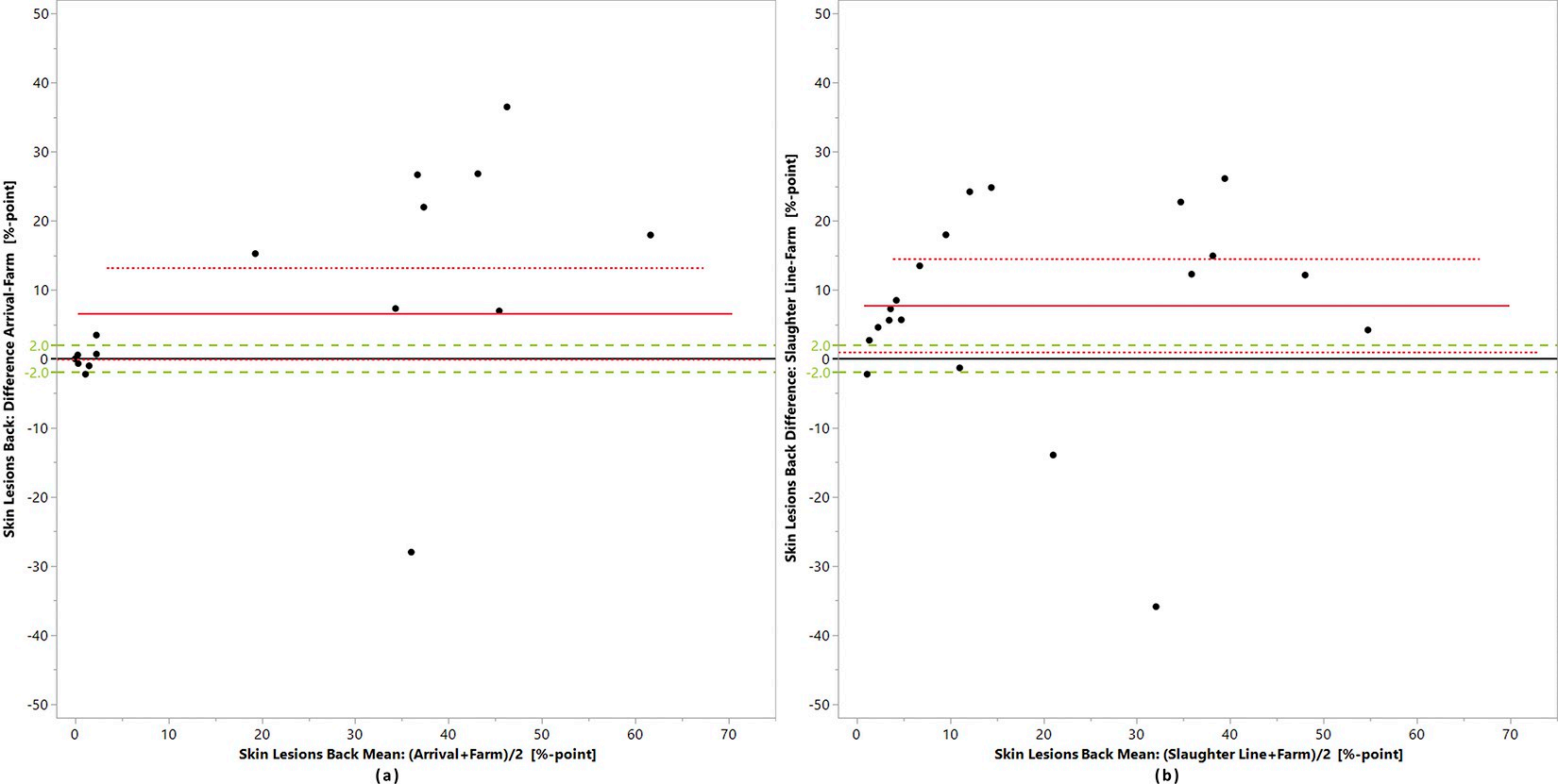
Prevalences of skin injury on the back. (a) on-farm versus slaughterhouse arrival and (b) on-farm versus slaughter line in 20 laying hen flocks; the black line is the zero line, the red line is the mean difference between the prevalences, the dotted red lines delimit the confidence interval of the differences and the green lines delimit the zone of equivalence, here set at +/- 2%-points.

**Fig 5:**
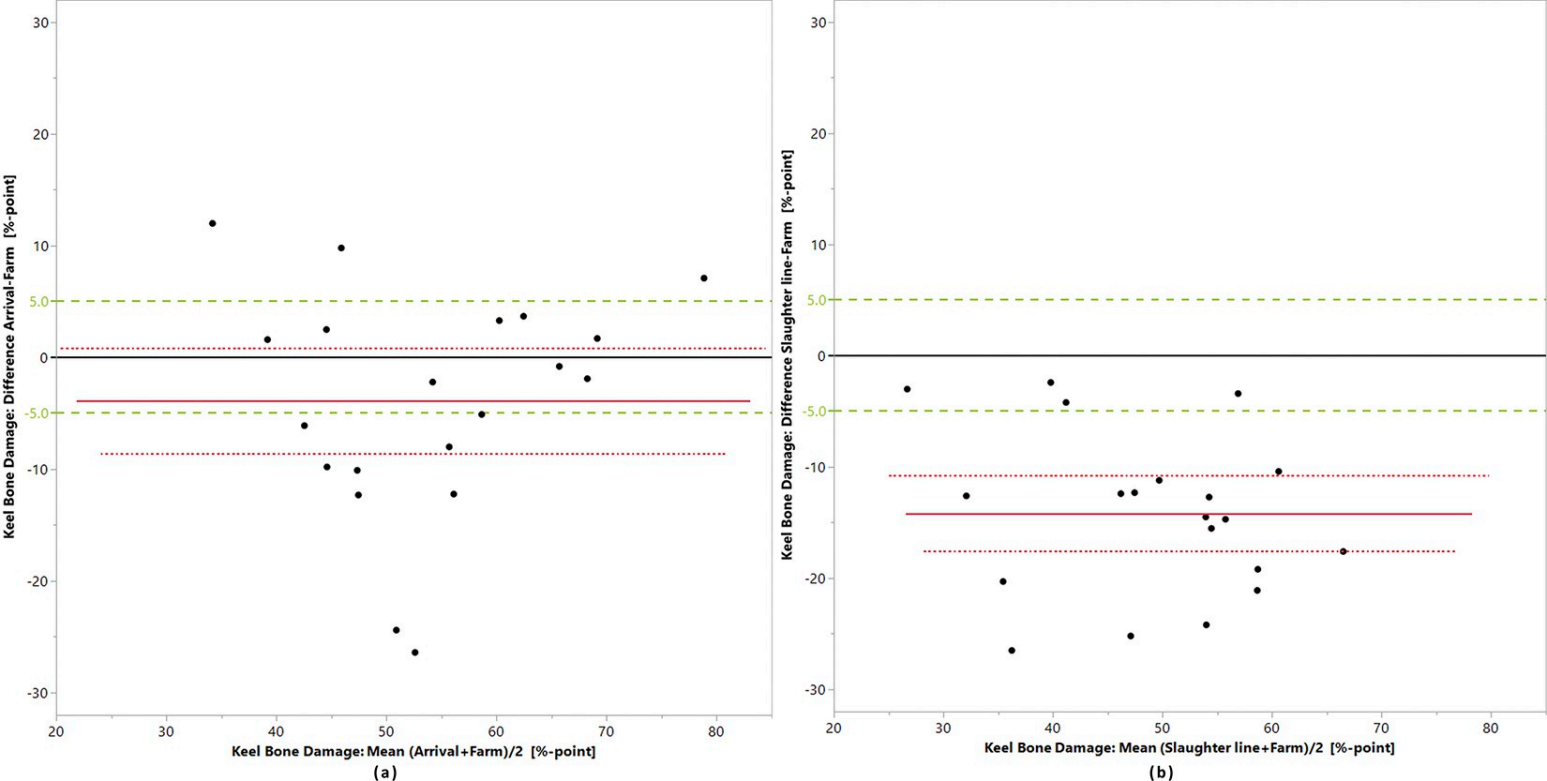
Prevalences of keel bone damage. (a) on-farm (score 1 and 2) versus slaughterhouse arrival (score 1 and 2) and (b) on-farm (score 1 and 2) versus slaughter line in 20 laying hen flocks; the black line is the zero line, the red line is the mean difference between the prevalences, the dotted red lines delimit the confidence interval of the differences and the green lines delimit the zone of equivalence, here set at +/- 5%-points.

Bland-Altman plots (Figs 3–5) and the equivalence tests (Table 6) show that for none of the indicators, the measurements at the different points of assessment fell within the predefined zones of equivalence.

**Table 6:** Results from the equivalence tests calculated with TOST for the prevalence comparison of skin injuries at the cloaca and back and for keel bone damage between the assessment time points on-farm, slaughter arrival and slaughter line.

Indicator	Equivalence comparison	Mean ≤ -2		Mean ≥ 2		See Fig
		t-ratio	*p*	t-ratio	*p*	
Skin injuries cloaca	On-farm vs. arrival	1.9582	0.0325*	-0.374	0.3564	4
Skin injuries back	On-farm vs. arrival	2.7117	0.0069*	1.4518	0.9186	5 (a)
Skin injuries back	On-farm vs. slaughter line	2.9983	0.0037*	1.7605	0.9528	5 (b)
**Indicator**	**Equivalence comparison**	**Mean ≤ -5**		**Mean ≥ 5**		**See Fig**
		**t-ratio**	***p***	**t-ratio**	***p***	
Keel bone damage	On-farm vs. arrival	0.4931	0.3138	-3.918	0.0005*	6 (a)
Keel bone damage	On-farm vs. slaughter line	-5.62	1.0000	-11.75	< .0001*	6 (b)

## Discussion

The aim of this study was to evaluate to which degree a monitoring of keel bone and skin status at the slaughterhouse can replace the on-farm laying welfare assessment regarding keel bone damage and skin injuries. Assessed on-farm, all 20 commercial laying hen flocks were affected by KBD with an overall average of 56.0% affected hens and nearly half of the flocks by skin injuries, with an overall average of 11.1% of hens with injuries at the cloaca region and an overall average of 15.1% at the back. Assessments at the slaughter line resulted in lower detected mean flock prevalences for KBD with 41.7%, but higher values for skin injuries at the back with nearly all flocks being affected at least slightly with an overall average prevalence of 22.8%. The latter is in line with results from Grafl et al. [27] who found skin damage on the back or rump in 95% of flocks with at minimum 1% and at maximum 25% affected hens. Similarly, the KBD prevalences found at the slaughter line conform to the results of Grafl et al. [27] who found 100% of flocks affected, with an average of 37% of the hens with KBD after defeathering.

Inter-observer reliability for skin injuries with a PABAK of on average 0.87 was comparable to results from other studies e.g. Freick et al. [34] and indicates that the method is suitable for the detection of skin injuries with respect to reliability. Conforming to Allain et al. [35] for cutaneous injuries in turkeys, reliability was slightly higher when skin injuries were assessed at the slaughter line compared to on-farm due their better visibility after defeathering. However, a clear differentiation between damage caused by the slaughter process and pecking or other injuries was not always possible, as will be further discussed below.

IOR values for KBD varied between substantial to almost perfect in case of trained persons, but was partly not substantial in case of untrained persons. It is therefore questionable if frequently changing slaughterhouse employees would be able to reliably score KBD. This need for proper and extensive training argues for advantages of an automated assessment that at the same time increases practicability, as very large sample sizes can be collected in a short period of time. A technical device allowing reliable assessments was developed and investigated by Jung et al. [26] and evaluated as very promising.

The comparison between the assessments on-farm and the arrival at the slaughterhouse was done to examine whether injuries might be attributed to catching and transport. This would mean that assessments at the slaughterhouse would not truly reflect the on-farm situation. The comparison between assessments on-farm and at the slaughter line in addition gave information about possible differences due to different assessment conditions and methods. For no indicator and comparison, equivalence within the set limits could be proven. Admittedly, the arbitrary limits can be discussed. When applying relatively low target values, like the 2% for skin injuries [28], an equivalence zone of +/- 2%-points appears reasonable. However, considering the commonly high prevalences of KBD, the applied +/- 5%-points for keel bone damage are debatable. Another possibility would be to take account of the confidence intervals of on-farm assessments with usually lower sample sizes due to feasibility issues. Our results show that the corresponding deviations between prevalence estimates can reach up to +/- 17%-points. Nevertheless, there were marked differences in the suitability of slaughter line recordings to replace on-farm assessments between skin injuries and KBD, which are discussed below. For the evaluated prevalences of skin injuries there was very low agreement between assessments on-farm and at the slaughter line and, additionally, changes from one evaluation range to another occurred into different directions, although we saw, as expected, a general numerical trend towards increasing numbers of hens with injuries from farm to slaughter line. Pecking events and other injury caused during catching, transport and during lairage could be a reason. Skin damage could additionally have been caused during the slaughter process or existing injuries became more visible after defeathering. Although in terms of feasibility, assessment at the slaughter line has the advantage of easier detection of alterations in higher sample sizes without extra handling of the hens, for the time being, we cannot confirm that injury prevalences recorded at the slaughter line reflect the on-farm status.

The assessment of skin injuries in the cloaca region was not feasible at the slaughter line. It is well established that cloacal cannibalism can occur independently from feather pecking and other forms of cannibalism [22]. Therefore, injuries on the back are not a good way to measure the general skin condition, and further technical means might be explored that allow assessments at the slaughter line. Probably the risk of damage being caused during the slaughter process is lower in the cloaca region than at the back. However, there was no equivalence between individual assessments and again changes between target, early warning or alarm ranges were not unidirectional, likely due to in part relatively low sample sizes (minimum of n = 31 at the arrival).

In contrast, the detected average KBD percentages of affected hens decreased from farm to arrival to the slaughter chain. The Bland-Altman plots show the consistent underestimation of KBD assessed at slaughterhouse arrival versus on-farm and at the slaughter line versus on-farm. The 30% of changes between the alarm and early warning ranges were also invariably unidirectional. Obviously, less damage can be assessed by visual inspection, in comparison to palpation. Surprisingly, the prevalences found on farm and at arrival were not equivalent. Responsible factors could be the lower sample size of hens evaluated at the arrival or negative influences on the assessor due to the need for rapid assessment and challenging environmental circumstances. Here the commercial framework conditions were partly limiting our study. The significant difference between prevalences on-farm and at the slaughter line were to be expected due to the only visual assessment after slaughter. However, due to the consistent underestimation of the slaughter line assessment vs. on-farm assessment, with a high correlation of *r* = 0.794, it appears acceptable to use slaughter line recordings, particularly for monitoring changes in prevalence, as long as it is recognized that true prevalences are higher than those recorded at the slaughter line, also taking into account the general underestimation of fractures in on-farm assessments without the help of radiographs. For the visual assessment of the carcass this is illustrated by the comparison with the inspection of pure bone, which is very close to assessments of x-ray images; only very small fissures might be missed. This comparison highlights a relatively low sensitivity of 70%, meaning that 30% of damaged bones could not be detected by visual inspection of the carcass. On the other hand, the high precision (93.33%) indicates that most of the bones identified as damaged were indeed damaged, suggesting a reliable identification of positive cases. Also, the specificity of 82.14% indicates a strong ability to correctly identify intact bones, with few false positives.

In conclusion, the high prevalences of skin injuries and KBD found on-farm, but also the considerable variation in flock prevalences, underline the need for a closer welfare monitoring. Although the assessment of welfare indicators at the “bottle neck” slaughterhouse cannot replace the assessment in the barn in terms of early warning, it may be a good control point for regular monitoring that might help to identify preventive or hazardous practices in the long term. The advantages in terms of feasibility of the assessment at the slaughter line appear not to be usable for skin injuries on the back. For injuries at the cloaca, further investigations on technical feasibility and reliability may be worthwhile. The assessment of keel bone damage at the slaughter line is easily possible concerning deviations. As deviation and fracture occurrence are correlated [13], assessing deviations at the slaughter line can give a rough picture of the current prevalence in the flocks. The consistent underestimation of real damage has to be considered when interpreting the results. Overestimation is unlikely; so it is a fairly robust indicator of changes in prevalence over time, though not a true measure of prevalence.

## Supporting information

S1 File (DOCX)
