# Supplementary material for: Can the assessment of skin injuries and keel bone damage at the slaughterhouse replace on-farm assessments?
Source: PLoS One. 2024 Dec 2;19(12):e0309137. doi: 10.1371/journal.pone.0309137 (PMC11611189; doi:10.1371/journal.pone.0309137)
Supplement: S1 File — (DOCX) [file pone.0309137.s001.docx]

Supporting Information

**Can the assessment of skin lesions and keel bone damage at the slaughterhouse tell about hen welfare on-farm? [Dataset]**

Link: <https://doi.org/10.48662/daks-30>
